# Supplementary material for: Overexpression of GhCAD6 in Upland Cotton (Gossypium hirsutum L.) Enhances Fiber Quality and Increases Lignin Content in Fibers
Source: Int J Mol Sci. 2025 Sep 29;26(19):9518. doi: 10.3390/ijms26199518 (PMC12524386; doi:10.3390/ijms26199518)
Supplement: Supplementary file 1 [file ijms-26-09518-s001.zip › ijms-3875162-supplementary-proofed.pdf]

CDS sequence of *GhCAD6* gene

ATGGGTAGCCTTGAAACTGAGAGAACAACCACAGGATGGGCTGCCAGAGATCCTTCAGGAGTATTGTCTCCTTACACTTACACTCTTAGGAACA
CTGGTCCTGAGGATGTTTTCGTTAAGGTTATGTGCTGTGGAATCTGCCACACTGATCTTCATCAAGCCAAAAATGATCTTGGCATGTCAAACTACC
CAATGGTTCCTGGGCATGAAGTGGTTGGTGAGGTGTTGGAAGTTGGGTCAGATGTAAGCAAGTTCAGAGTTGGTGATATTGTTGGTGTTGGTTGT
CTTGTTGGATGTTGCAGAAACTGCCGACCATGCGACTCAGACAATGAACAATACTGTCTCAAGAAGATCTGGTCGTACAATGATGTTTACACTGA
CGGCAAACCCACCCAAGGTGGCTTTGCTGGTTCCATGGTCGTTGATCAAAAGTTTGTGGTGAAAATCCCAGAAGGAATGGCACCAGAACAGGT
GGCACCGCTGTTATGTGCAGGGGTGACAGTTTACAGCCCATTGAATCACTTTGGTTTAATGGGGAGTGGGTTAAGGGGAGGAATATTGGGACTTG
GAGGTGTAGGGCACATGGGGGTGAAGATAGCCAAAGCAATGGGGCATCACGTAACAGTTATAAGCTCATCTGATAAGAAAAAAGTGGAGGCTTT
GGAGCATCTTGGTGCTGATGACTACTTAGTCAGCTCCGACGCTGAAGGGATGCAAAAGGCTGCTGATTCACTTGATTATATCATTGACACTGTGC
CTGTTTTTCACCCACTTGAGCCTTACCTTTCATTGTTGAAATTCGACGGCAAGTTAATCTTGACTGGTGTTATTAACACCCCTCTTCAGTTTGTTTC
CCCTATGGTCATGCTTGGGAGAAAGTCGATTACAGGGAGTTTTATTGGGAGCATGAAGGAAACAGAGGAAATGCTTAACTTCTGTAAGGAGGAA
AATTTGACCTCAATGATTGAGGTGGTGAAGATGGATTATATCAACACAGCAATGGAGAGGCTGGAGAAGAACGATGTTCGATACAGATTCGTCGT
GGATGTTGCTGGAAGCAAGCTTGATCAATAG

Table S1 List of primer sequences

Primer name	Primer sequence	Note
NPT II-F	CATGGGTCACGACGAGATCA	Positive plant detection
NPT II-R	ATGACTGGGCACAACAGACA	
E6-F	CGCAATTCCACATCACACAC	
GhCAD6-R	TCATTGTCTGAGTCGCATGG	

Table S2 Comparison of fiber quality parameters between overexpression plants and control plants

Lines	Fiber upper half mean length (mm)	Fiber uniformity index (%)	Fiber stength (cN/tex)	Boll number per plant	Micronaire (μg)	Lint (%)
CK	26.71±0.39	84±0.98	24.9±0.28	5.5±0.71	4.93±0.39	41.6±0.0027
OE-*GhCAD6*#1	31.28±0.28	85.63±0.75	30.93±0.5	8.33±1.53	4.26±0.38	43.6±0.0025
OE-*GhCAD6*#2	30.05±0.09	86.33±0.35	29.07±0.25	10.67±1.15	4.8±0.05	44.6±0.0056
OE-*GhCAD6*#3	30.2±0.56	84.93±0.67	29.27±0.55	9.33±0.58	4.54±0.4	43.0±0.0012
OE-*GhCAD6*#4	28.01±0.59	83.7±0.7	26.37±0.5	10.33±1.53	4.5±0.05	44.3±0.0016

The values are the average value of CK (n = 2) ± SD and the average of transgenic lines (n = 3) ± SD.
